# Harmonized Exposome Data for Aging Populations Within the Gateway Data Sharing Platform

**DOI:** 10.1093/geroni/igaf122.042

**Published:** 2025-12-31

**Authors:** Sara Adar

**Affiliations:** University of Michigan School of Public Health, Ann Arbor, Michigan, United States

## Abstract

The Gateway to Global Aging Data has harmonized environmental exposome measures for seven nationally representative aging cohorts across the Americas, Europe, and Asia. Through a collaboration across studies within the Health and Retirement Study International Network, cohorts have been linked to information about air pollution (i.e., total and source-specific fine particulate matter, nitrogen dioxide, and ozone), weather (i.e., temperature and heat), and the built environment (i.e., greenspace and blue space) allowing for the examination of risk and protective factors for health. These cohorts are committed to providing data access to the scientific community, opening new opportunities for innovative research on the environmental exposome and health in aging populations. In addition to promoting data availability, the Gateway project has also conducted methodological research on topics such as spatial confounding and data security as well as developed comprehensive user guides to support studies on the exposome and aging outcomes. This presentation will showcase these powerful resources and demonstrate how researchers can leverage them to advance science.

